# Design and Simulation of a Ring Transducer Array for Ultrasound Retinal Stimulation

**DOI:** 10.3390/mi13091536

**Published:** 2022-09-16

**Authors:** Chenlin Xu, Gengxi Lu, Haochen Kang, Mark S. Humayun, Qifa Zhou

**Affiliations:** 1 Department of Biomedical Engineering, University of Southern California, Los Angeles, CA 90089, USA; 2 USC Roski Eye Institute, Keck School of Medicine, University of Southern California, Los Angeles, CA 90033, USA; 3 USC Ginsburg Institute for Biomedical Therapeutics, University of Southern California, Los Angeles, CA 90033, USA

**Keywords:** ultrasound, retinal prosthesis, neural stimulation, array transducer, visual restoration

## Abstract

Argus II retinal prosthesis is the US Food and Drug Administration (FDA) approved medical device intended to restore sight to a patient’s blind secondary to retinal degeneration (i.e., retinitis pigmentosa). However, Argus II and most reported retinal prostheses require invasive surgery to implant electrodes in the eye. Recent studies have shown that focused ultrasound can be developed into a non-invasive retinal prosthesis technology. Ultrasound energy focused on retinal neurons can trigger the activities of retinal neurons with high spatial-temporal resolution. This paper introduces a novel design and simulation of a ring array transducer that could be used as *non*-invasive ultrasonic retinal stimulation. The array transducer is designed in the shape of a racing ring with a hemisphere surface that mimics a contact lens to acoustically couple with the eye via the tear film and directs the ultrasound to avoid the high acoustic absorption from the crystalline lens. We will describe the design methods and simulation of the two-dimensional pattern stimulation. Finally, compared with other existing retinal prostheses, we show that the ultrasound ring array is practical and safe and could be potentially used as a *non*-invasive retinal prosthesis.

## 1. Introduction

The human retina is a thin and light-sensitive layer of tissue located inside the eye. When the two-dimensional image light of the visual world passes through the photoreceptor layer of the retina, it converts into patterned electrical impulses, which then go through optic nerves to the brain. Thus, the visual sensation is generated in the visual cortex [1]. Inherited retinal degenerative diseases such as retinitis pigmentosa and age-related macular degeneration are the leading cause of blindness [2,3,4]. These diseases induce deterioration of photoreceptor cells, which cause irreversible loss of vision [5,6]. Retinal prostheses based on electrical stimulation of remaining retinal neurons such as bipolar cells or retinal ganglion cells (RGCs) have been developed and shown to restore some level of vision to otherwise blind patients who have lost their photoreceptors [7,8,9]. One example of such a device is the Argus II Retinal Prosthesis System (Second Sight Medical Products, Inc., Sylmar, CA, USA) that has obtained approvals from the US Food and Drug Administration (FDA) in 2013 [10]. It consists of a camera in a glass frame that captures the visual image in real-time, a video-processing unit (VPU), an external radiofrequency (RF) coil, and a 6 × 10 round, rectangular microelectrode array (MEA) implanted in the retina and stimulates the bipolar cells [7,11]. The energy required of electrical stimulation is generated with an RF link between external electronics and implanted MEA [11]. Although it has been in clinical use, the fabrication process of such devices is challenging, including engineering a hermetic seal for the implanted electronics and electrode material that can withstand chronic electrical pulses. In addition, these devices require surgical implantation and are expensive [12].

Several non-invasive neuromodulation methods have been explored to overcome the challenges of high-risk invasive surgery in brain research, such as transcranial magnetic stimulation (TMS) and transcranial direct current stimulation (tDCS) [13]. For retinal stimulation, however, these stimulation methods have suffered from poor spatial resolution (on the scale of centimeters). In contrast, retinal stimulation typically needs resolution at the micron scale targeting retinal neurons [14,15,16]. Thus, both techniques are not suitable for retinal stimulation. Another approach is based on optogenetic methods [17,18,19]. The stimulation could achieve single-neuron resolution by genetically modifying the specific cell desired to respond to light. Although a few clinical trials have been reported so far [20], the ethical issues regarding human trial of genetic modification would rise challenge for widespread clinical application.

Focused ultrasound neurostimulation (FUS) based on ultrasonic transducer has drawn increased attention as a non-invasive neuromodulation technique in recent years because of its ability to pass through human tissue non-invasively and directly trigger neural spikes [15,21]. Several in-vitro and in-vivo studies of ultrasonic neuromodulation in various animal models such as mice [21,22,23], rabbits [24], rhesus macaque [25], and humans [26,27] have shown great promise to develop the next-generation, non-invasive acoustic retinal prosthesis by using ultrasound to stimulate the retina with the application-specific transducer directly. In 2013, Menz et al. [28] used a single-element ultrasonic transducer that was able to create high-frequency FUS (43 MHz) to stimulate isolated salamander retina with a better spatial resolution (90 μm spots) and temporal precision. 

The array-based ultrasonic transducer is the next-generation device for ultrasound neuromodulation. With 3D printing technology, the properties of piezoelectric materials can be precisely controlled [29]. Unlike single-element ultrasonic transducer, where the stimulation point is mechanically fixed, the array-based ultrasonic transducer can stimulate multiple points simultaneously using transmission beamforming technology [30,31], thus enabling two-dimensional pattern stimulation with a multi-focus ultrasonic field. For imaging applications, an array transducer can also reduce inspection time and improve the quality of images [32]. In 2012, Naor et al. reported the first acoustic retinal prosthesis with ultrasound. They used a multi-element ultrasonic phased array with an external camera and an integrated image processor to generate a focused, two-dimensional image pattern. They showed that the acoustic retinal prosthesis is capable of directly stimulating visual evoked potentials (VEPs) in the RGCs of Sprague Dawley rats with low-frequency FUS (0.5 MHz and 1 MHz) [33,34]. Later in 2017, Gao et al. [35] proposed a simulation study of a multi-element transducer array that mimics the contact lens shape of the human eye. However, the high acoustic absorption (7.8 dB @ 10 MHz) [36] of the lens tissue of the human eye can attenuate the stimulation effect of ultrasound and further damage the eye due to the energy absorption in the lens. We have proposed a possible method to overcome this issue by making the transducer array flexible and hollow in the center [37]. Although the flexible and concave shape of the array facilitates the acoustic coupling with the tear film, variation of the location of each transducer element would cause the difficulty of beamforming and thus arise the echo noise due to the stretchable nature of the array. In addition, the center frequency of the array transducer of this work is 5 MHz, which is too low to meet the spatial resolution requirement for precise retinal stimulation.

In this work, a novel phased array for ultrasound retinal stimulation is proposed. The center frequency of the array transducer is designed as 20 MHz. The array transducer is designed in the shape of an oval edge with a ring hollow in the center to fit with the shape of the human eye and bypass the acoustic absorption of lens tissue due to the high frequency. The interface of the array is designed as a hemisphere surface to provide intimate contact between the transducer and eye liquid. Each transducer element is expected to be aligned along the hemisphere surface. The ultrasonic wave could reach retinal tissue in-depth to generate high-quality multifocal stimulation. The simulation of the two-dimensional pattern generation is also presented as a theoretical guide to improving the device’s performance.

## 2. Methods

### 2.1. Strategy of FUS Retinal Stimulation

**Figure 1:**
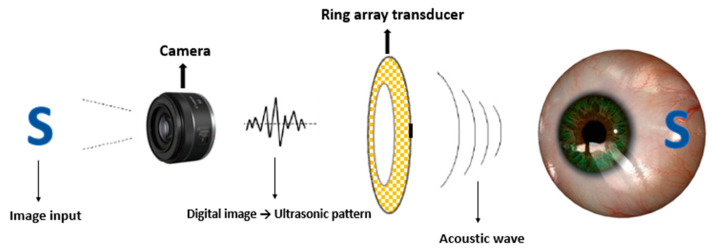
Concept of an ultrasonic stimulated retinal prosthesis. (Image reference from Lo PA et al., 2020 [38]. Reproduced with permission from from Lo, Pei-An et al., Micromachines; published by Lo, Pei-An et al., 2020).

**Figure 2:**
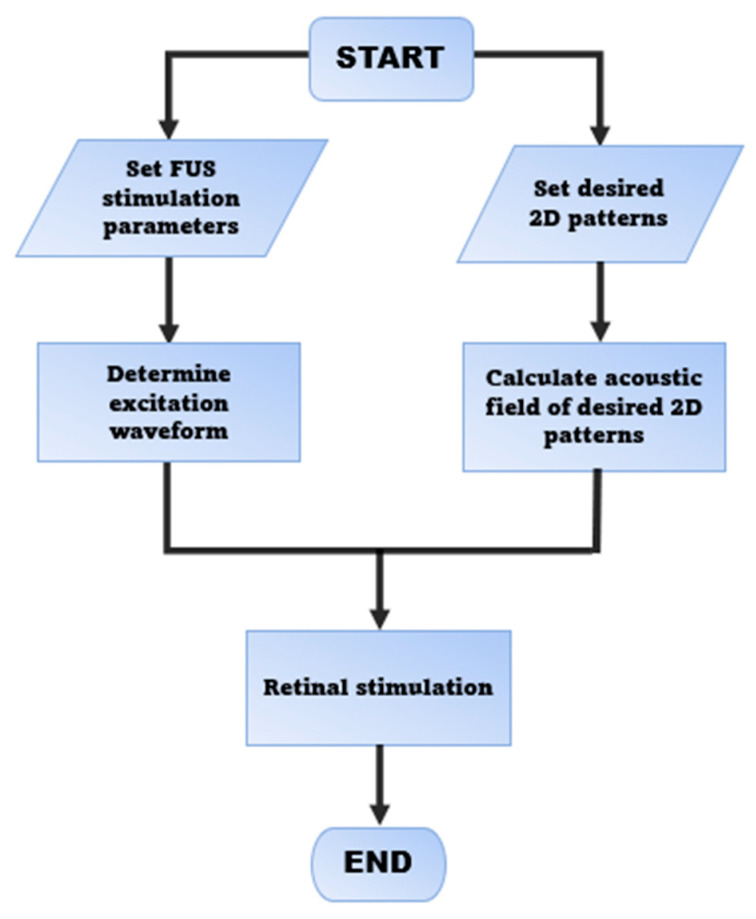
Strategy of ultrasonic retinal stimulation.

The concept and strategy for FUS retinal stimulation is demonstrated in Figure 1 and Figure 2, respectively, which are similar as what we proposed previously [38]. Our FUS retinal stimulation strategy is as follow. First, the desired image or visual scene will be first captured by an external camera and then processed into a digital image as the input signal. Second, digital input signal will be further processed into the same acoustic pattern field via the 2D Fourier transform technique, and finally, acoustic pattern field will be transferred into the retina through array transducer that enable retinal stimulation.

### 2.2. Design of the Ring Array Transducer

**Figure 3:**
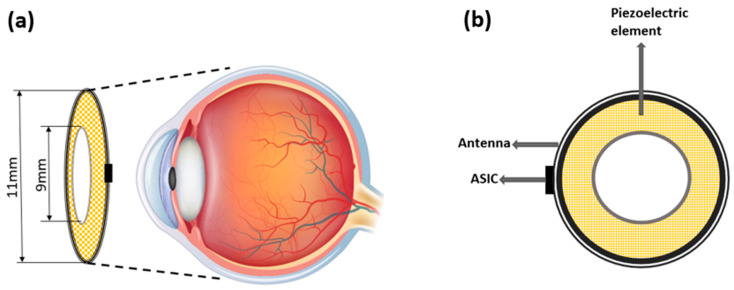
Configuration of racing ring array transducer for retinal stimulation. (**a**) End-view of the proposed device. (**b**) Top view of the proposed device. (Image reference from Yu et al., 2019 [37]. Reproduced with permission from from Yu, Yanyan et al., Seneors; published by Yu Yanan et al., 2019).

The design configuration of the racing ring array in shape and size is demonstrated in Figure 3a. The array transducer is designed as the racing ring. The outer diameter (OD) and inner diameter (ID) of the ring are designed to be 11 mm and 9 mm, respectively. The circular hollow in the inner ring could bypass the lens tissue. The radius curvature of the racing ring is designed to be 12 mm, which matches the size of an adult human eyeball [31].

The proposed scheme of completing the device is demonstrated in Figure 3. The array transducer is designed as racing ring. The camera that captured the visual scene can be integrated into a commercially used goggle, and the racing ring array can be placed on the surface of the human cornea non-invasively. In addition, the acoustic gels can also be applied to provide intimate contact between the array and cornea to minimize the reflection of the ultrasound [37,38]. The device is wirelessly powered by an external RF signal with the antenna. An application specific integrated circuit (ASIC), placed near the interface between array transducer and antenna, can be used to process the signal and drive the racing ring array transducer.

The design parameters of our proposed array transducer are listed in Table 1. Based on our previous simulation results [37], the number of elements is chosen as 512 with a 0.075 mm (1 λ) element pitch. The center frequency is selected as 20 MHz. 

### 2.3. Simulation of the Ultrasonic Excitation Waveform and Pattern

#### 2.3.1. Determine the Excitation Waveform of Each PZT Element

**Figure 4:**
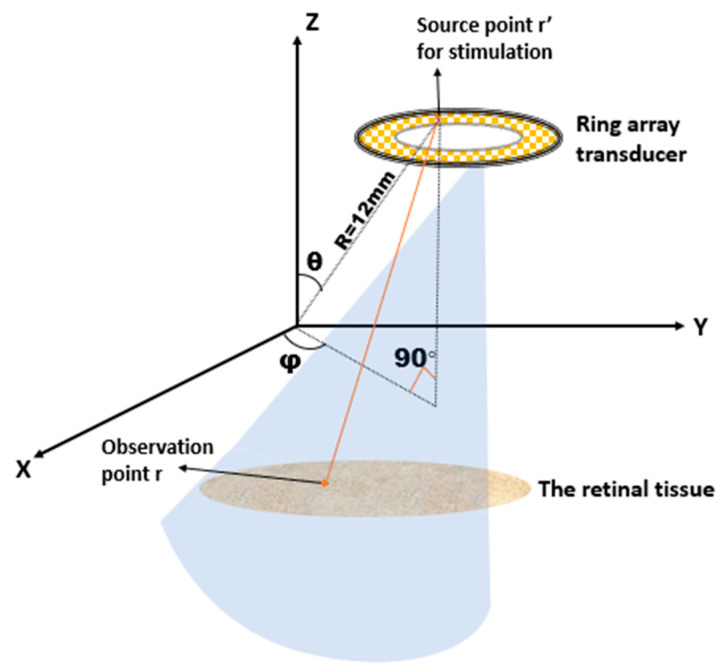
Scheme for calculating the ultrasound field by racing ring array (Image reference from Yu, Y et al. [37] Reproduced with permission from from Yu, Yanyan et al., Seneors; published by Yu Yanan et al., 2019).

The simulation of a 2D pattern of acoustic pressure field generated in a racing ring surface is performed using the method proposed by Ebbing et al. [31] based on discretized Rayleigh–Sommerfeld diffraction integrals. The acoustic pressure P on point r of an ultrasonic waveform from a source point r′ could be obtained by:

(1) $$P\left(r\right)=\frac{j\rho ck}{2\pi }\int u\left(r^{\prime }\right)\frac{\exp\left(-jkd_{rr^{\prime }}\right)}{d_{rr^{\prime }}}dS$$

where P(r) is the pressure of the ultrasound at the observation point, ρ is the density of the medium, c is the propagation velocity of ultrasound in the medium, k is the number of ultrasonic waves, u(r′) is the velocity of the excitation waveform at point r′ on the surface of the source point of the transducer array, drr′ is the distance between r and R, and S is the surface area of the power source of ultrasound. We assume the number of elements in our racing ring transducer array is N, and each transducer element produces a spherical waveform with an amplitude positively proportional to the area of its surface; the acoustic pressure of the observation point r can be described as

(2) $$P\left(r\right)=\frac{j\rho ck}{2\pi }\overset{N}{\underset{n=1}{\sum }}u_{n}\int \frac{e^{j\left(wt-k\left|r-r_{n}^{'}\right|\right)}}{\left|r-r_{n}^{'}\right|}dS_{n}$$

where $u_{n}\;$ is the velocity of the excitation waveform of the n^th^ element, $r_{n}^{'}$ is the source points on the n^th^ element, and $dS_{n}$ can be rewritten as:

(3) $$dS_{n}=R^{2}sin\varphi d\varphi d\theta$$

where R is the radial distance from the array transducer, θ is the azimuthal angle, and φ is the polar angle. Figure 4 shows their position on the spherical coordinate system 

Therefore, based on Equation (3), the acoustic pressure at the mth observation point can be calculated as:

(4) $$P\left(r_{m}\right)=\frac{j\rho ck}{2\pi }\overset{N}{\underset{n=1}{\sum }}u_{n}\iint \frac{e^{j\left(wt-k\left|r_{m}-r_{n}^{'}\right|\right)}}{\left|r_{m}-r_{n}^{'}\right|}dS_{n}\;$$

The term $\frac{j\rho ck}{2\pi }\iint \frac{e^{j\left(wt-k\left|r_{m}-r_{n}^{\prime }\right|\right)}}{\left|r_{m}-r_{n}^{\prime }\right|}$ in the above equation could be replaced by forward propagation operator H, that is, H = $\frac{j\rho ck}{2\pi }\iint \frac{e^{j\left(wt-k\left|r_{m}-r_{n}^{\prime }\right|\right)}}{\left|r_{m}-r_{n}^{\prime }\right|}$. In addition, let U = [u_1_, u_2_, u_N_] as the vector that describes the velocity of excitation waveform of N elements of the transducer array. Together, the Equation (4) could be rewritten as:

(5) P=HU

To solve the excitation term U, the minimum norm least-square method is used [39] as follows:

(6) $$\hat{U}={\left(H^{*}\right)}^{t}{\left(H{\left(H^{*}\right)}^{t}\right)}^{-1}$$

The iterative weighting method [37,39] is used to improve the efficiency by:

(7) $$\hat{U}=W{\left(H^{*}\right)}^{t}{\left(HW{\left(H^{*}\right)}^{t}\right)}^{-1}$$

where W is the weight that could be written as an N × N positive matrix, which could be calculated by an iterative weighting algorithm [40]

#### 2.3.2. Determine the Pattern Generation Algorithm 

**Figure 5:**
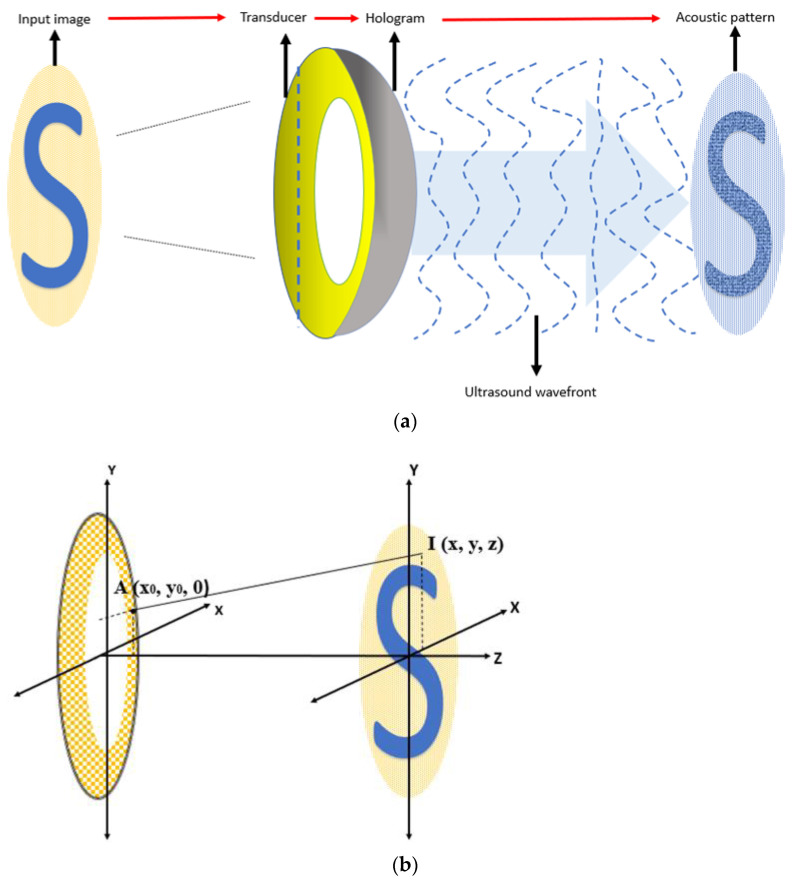
(**a**) Workflow for generating acoustic pattern with given image. (**b**) Image input (on the right) and the corresponding aperture function (on the left) in the coordinate system (Image reference from X Wu et al. [39] and Melde, K et al. [42]).

Continuous and uniform 2D ultrasound field pattern was simulated by weighted Gerchberg–Saxton (GSW) iterative algorithm and angular spectrum field calculations [34,41] based on the Fourier relation between ultrasonic source plane (back) and target plane (front). The workflow for generating desired acoustic field pattern based on targeted image is as Figure 5a. First, image will be captured by an external camera and further processed as the same size of the array source plane. The amplitude of the input image will be the input of GSW iterative algorithm. Then, the hologram plane will convert ultrasound wavefront from array transducer to the phase plane, and finally this travelling ultrasound wave will form the final acoustic pattern. We assume the target plane is placed in the far-field of the array transducer. The relation between the pattern of the image in coordinate system I (x,y) and aperture function of our array transducer A is obtained by 2D Fourier transform:

(8) $$I(x,y,z)=\frac{1}{\lambda z}F\left\{A\left(x_{0},y_{0}\right)\right\}{|}_{u=\frac{x}{\lambda z}\;,\;\;v=\frac{y}{\lambda z}\;\;}$$

Therefore, based on Equation (8), the aperture function of the transducer could be obtained by 2D inverse Fourier transform once the pattern from the digital image is provided:

(9) $$A\left(x_{0},y_{0}\right)=\frac{1}{\lambda z}F^{-1}\left\{I(u,v,z)\right\}{|}_{x=\frac{x_{0}}{\lambda z}\;,\;\;y=\frac{y_{0}}{\lambda z}\;\;}$$

The discrete version of algorithm of Equation (9) that compute the excitation waveform of each element is as follows:

(10) $$A\left(x_{0},y_{0}\right)=\sum_{m=1}^{M}\sum_{n=1}^{N}I\left(m,n\right)\exp\left(j2\pi (u_{m}\frac{x_{0}}{\lambda z}+v_{n}\frac{y_{0}}{\lambda z})\right)$$

Based on Equation (10), the desired pattern can be projected given an arbitrary input digital image. The relationship of image input and its corresponding aperture function of the transducer in the coordinate system is shown in Figure 5b.

#### 2.3.3. Determine the Ultrasonic Frequency for Stimulation and Imaging

The spatial resolution and stimulation efficacy are dependent on the acoustic frequency of the ultrasound and have received little systematic consideration so far. The spatial resolution of ultrasonic neuro-stimulation is determined by the acoustic wavelength λ and F-number of the system [34], the ratio between the distance from the array to the focal plane, and the effective aperture of each element of the array. In general, the intensity in the location of full width at half the maximum (FWHM), the width of ultrasound spectrum curve at half of the maximum point, is used as a measurement of the maximal stimulation efficacy:

(11) $$D_{l}=K\lambda F_{\#}=K\frac{v}{f}F_{\#}$$

where $D_{l}$ is the intensity at FWHM, K is a constant value mostly equal to 1,$\;F_{\#}$ is the F-number of the system, f is the frequency of the ultrasound, and v is the speed of sound in the medium, in this case, the retinal tissue area. Based on Equation (11), we can place our array transducer as near as possible to the cornea and increase the aperture so that $F_{\#}$ is decreased and thus improve the spatial resolution.

In addition, the use of high-frequency ultrasound can also improve spatial resolution. Previous research reported the sub-mm spatial resolution could be achieved by using sub-MHz high acoustic frequency stimulation from 2.3 MHz [34], 4.04 MHz [39], and 43 MHz [28], but higher frequency requires the corresponding high acoustic intensity to maintain the stimulation efficacy. Here, ultrasonic stimulation efficacy was obtained by the bilayer sonophore model [34,43]. In this model, the maximal areal strain of the bilayer membrane leaflets $\varepsilon_{max}$ is used to measure the stimulation efficacy as follows:

(12) $$\varepsilon_{max}\propto \;\frac{P_{A}^{\beta }}{\sqrt{f}}$$

(13) $$P_{A}\propto f^{1/2\beta }$$

where $P_{A}$ is the negative peak pressure of ultrasound and β is a constant range from 0.8 to 0.9 [43]. Based on the above equations, the power intensity of ultrasound is expected to increase proportionally to $f^{1/2\beta }$ to achieve the same stimulation efficacy. However, in the retinal tissue, ultrasound will attenuate depending on its frequency, denoted as:

(14) $$a=a_{0}f^{\gamma }$$

where α is used to predict the FUS attenuation, $a_{0}$ is the coefficient of FUS attenuation at 1 MHz ultrasound, and γ is around 1.0 to 1.9 [44,45]. 

## 3. Result

### 3.1. Acoustic Power Distribution 

**Figure 6:**
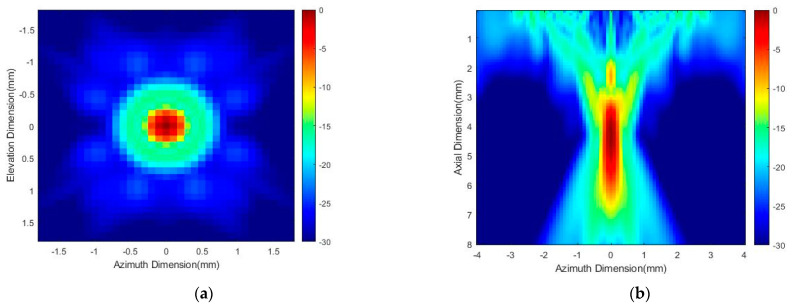

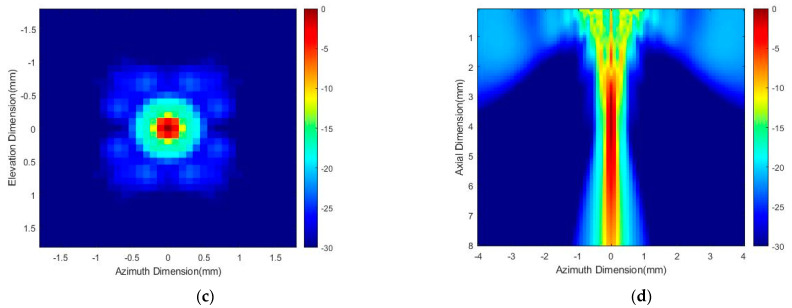
(**a**)Distribution of acoustic field in the XY plane in 5 MHz. (**b**) Distribution of acoustic field in the XZ plane in 5 MHz. (**c**) Distribution of acoustic field in the XY plane in 20 MHz. (**d**) Distribution of acoustic field in the XZ plane in 20 MHz.

Distribution of acoustic field of the racing ring array transducers with center frequency of both 5 MHz and 20 MHz were simulated using Field II (version 3.30) computational platform with MATLAB (R2020a, Mathworks, Natick, MA, USA) software. Figure 6a,c demonstrate the simulation result of acoustic field power distribution in the 1.5 mm × 1.5 mm lateral planes (X-Y plane) with 5 MHz and 20 MHz array transducer, respectively. The stimulation depth in this simulation is 7 mm for retinal stimulation of the rat retina. The lateral focal width of the transducer at −3 dB was around 1.5 mm. Figure 6b,d demonstrate the simulation result of an acoustic field power distribution in the axial plane (X-Z plane) with 5 MHz and 20 MHz array transducer, respectively. The lateral focal width at −3 dB was around 1.5 mm, which is the same as the lateral plane.

### 3.2. Pattern Generation Result

**Figure 7:**
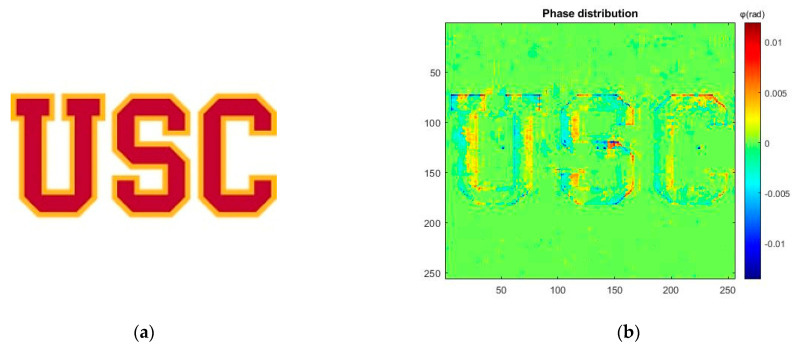

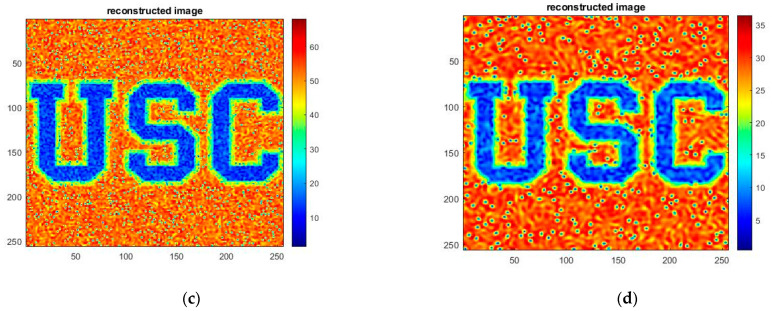
(**a**) Image input with 256 × 256 pixels. (**b**) Phase distribution (ϕ) for 5 MHz acoustic field. (**c**) Pattern distribution of acoustic field in the XY plane in 5 MHz. (**d**) Pattern distribution of acoustic field in the XY plane in 20 MHz.

Figure 7a,b shows the letter “USC” as the inputs image with 256 × 256 pixels of our system [46] and the corresponding phase diagram of input image as a hologram, respectively. Figure 7c,d show the acoustic field power distribution pattern with 5 MHz and 20 MHz, respectively. The dimension of output pattern is 8.96 mm × 8.96 mm which is match with the size of input image. Each pixel is corresponding to half lambda wavelength of 20 MHz ultrasonic wave. The weighted GSW algorithm is used to generate the pattern with the excitation acoustic field presented in the last section. 

## 4. Discussion

In this paper, we proposed design strategy and simulation of a novel racing ring array transducer for non-invasive retinal stimulation of rat. The proposed array transducer can be placed on the eye’s anterior surface like a contact lens. The inner hollow allows the ultrasound to bypass the lens tissue to avoid the high acoustic absorption [36]. 

The frequency of ultrasound of stimulation of both 5 MHz and 20 MHz were simulated, and the depth of ultrasound could reach in this simulation study is chosen as 7 mm to match the anatomical structure of adult rat’s retina. Compared to our previous work using 5 MHz ultrasound [37], 20 MHz high-frequency ultrasound can increase the spatial resolution. The inner hollow design enables the ultrasonic wave to bypass the lens tissue, thus avoid acoustic attenuation and the ultrasound can reach the retinal tissue deeper, which improves the stimulation efficacy. In addition, since the wave-length is inversely proportional to the ultrasound frequency, higher-frequency ultra-sound requires a smaller element size. Previous research shows that the total number of elements of the array would be more than several thousand if high-frequency ultra-sound is chosen [35]. For this reason, lower-frequency ultrasound is more feasible for practical applications. However, lower frequency ultrasound sacrifices the spatial resolution. For retinal stimulation, lower frequency ultrasound can easily stimulate the nearby retinal neuron of our target neuron, which would decrease the stimulation efficacy. To overcome these challenges, it is important to further explore more efficient ultrasonic stimulus waveform (i.e., center frequency, duration, amplitude) to achieve cell-type specific retinal stimulation. 

FDA regulation [37,47] has assigned the acoustic intensity for the eyeball used in clinics should be lower than 50 mW/cm^2^. Based on this regulation, our design is expected to require 80.3 mW for the in total 8.96 mm × 8.96 mm pattern area, which is safer than conventional ultrasound transducers because of its lens-avoiding structure, but we still need to conduct further studies to confirm these safety findings. The design of our ring array transducer can bypass the lens tissue so the intensity level of the ultrasound can be higher while still achieving the same stimulation efficacy. Despite all that, the ultrasonic retinal prosthesis is still in its infancy due to the unclear biophysical mechanism. For instance, recent studies also showing that ultrasound-induced neural activities can result from combinational mechanisms instead of single mechanism, such as capacitive current generated from displacement of neural membrane during ultrasonic stimulation, activation of mechanosensitive ion channels, sonoporation, and mechanical coupling when ultrasonic waves propagate along the axon [48]. In this simulation, we assumed the neural activities are generated by the capacitive current as it is accordance with both recent studies where membrane capacitive changes and subsequently the generation of action potentials were discovered [48], and the well-established Hodgkin and Huxley model [49] where neural membrane also modelled as capacitive components. Future direction will also include determine the exact bio-physical mechanism of ultrasound neuromodulation to optimize the ultrasonic stimulus. 

## 5. Conclusions

A novel design and the simulation of racing ring array transducer are proposed for the development of next-generation non-invasive ultrasound retinal prosthesis. We demonstrated an optimized FUS retinal stimulation strategy with 20 MHz high-frequency ultrasound based on the proposed array transducer. The racing ring design, as a contact lenses, enables the ultrasound bypasses the eye lens so that it can reach deep retinal tissue and avoids the high acoustic absorption, and by doing so, the stimulation efficacy can be significantly improved. This study provides theoretical guidance for the next step of fabricating acoustic retinal prosthesis.

## Figures and Tables

**Table 1:** Transducer designed parameters.

Designed Parameter	Value
Array Dimensions	OD = 11 mm, ID = 9 mm
Center Frequency	20 MHz
Number of Element	512, 10% sparse
Element Size	0.15 × 0.15 mm^2^
Element Pitch (1 λ)	0.075 mm
Thickness	1.4 mm
Stimulation Depth	7 mm
Radial Curvature	12 mm

## Data Availability

Data generated at a central, large-scale facility, available upon request.
